# Sex differences in skeletal muscle size, function, and myosin heavy chain isoform expression during post‐injury regeneration in mice

**DOI:** 10.14814/phy2.15791

**Published:** 2023-08-24

**Authors:** Jae‐Sung You, Pallob Barai, Jie Chen

**Affiliations:** ^1^ Department of Cell and Developmental Biology University of Illinois at Urbana‐Champaign Urbana Illinois USA; ^2^ Department of Bioengineering University of Illinois at Urbana–Champaign Urbana Illinois USA; ^3^ Nick J. Holonyak Micro and Nanotechnology Laboratory University of Illinois at Urbana–Champaign Urbana Illinois USA

**Keywords:** cross‐sectional area, fatigability, force

## Abstract

Skeletal muscle regeneration is an essential process to restore muscle function after injury and is influenced by various factors. Despite the known importance of sex hormones in muscle regeneration, whether and what sex difference exists in this process is still unclear. In this study, we provide evidence for a clear sex difference in muscle regeneration in mice. At 7 and 14 days after barium chloride‐induced muscle injury, female mice showed a faster recovery of muscle fiber size than males. Consistently, muscle force in female mice was restored faster than in males after injury, and this functional difference was maintained at 14 months of age when regenerative capacity declined. Myosin heavy chain isoform profiling and fatigability test revealed dynamic remodeling of myosin heavy chain isoform expression including a type IIB to IIA/X MHC transition and reduced fatigability in regenerated muscles compared to uninjured muscles. A significant sex difference was detected in myosin heavy chain IIX content, although this did not lead to different fatigability. Together, our results suggest that sex is an important determinant of the recovery of regenerating skeletal muscle and is partially involved in the remodeling of myosin heavy chain isoforms during muscle regeneration.

## 1 INTRODUCTION

Skeletal muscle can be damaged by various physical and chemical stresses in healthy conditions, but it can occur more severely and frequently in some disease conditions, such as dystrophin deficiency and aging (Brooks & Faulkner, 1996; Dellorusso et al., 2001). Upon severe necrotic damage, skeletal muscle undergoes a robust regeneration process to restore normal muscle function through muscle stem cell (MSC) activation, proliferation, and differentiation of myoblasts, as well as timely modulation of these steps by muscle cell‐intrinsic and ‐extrinsic factors (Forcina et al., 2020; Son et al., 2019). Among cell‐extrinsic factors, sex hormones have been shown to play an essential role in muscle regeneration. For example, the combined use of ovariectomy and exogenous estrogen replacement in rodents has demonstrated that 17β‐estradiol, likely through estrogen receptor‐β, promotes MSC activation and regeneration following myotoxin‐induced acute injury (Chaiyasing et al., 2021; Velders et al., 2012). Likewise, testosterone supplementation in castrated mice has been shown to increase MSC proliferation and the cross‐sectional area (CSA) (i.e., size) of regenerating myofibers (Serra et al., 2013).

Although the critical role of female and male hormones in fskeletal muscle regeneration is well recognized, whether regeneration efficiency differs between the two sexes has yet to be resolved. For example, Deasy et al. showed that, when transplanted into dystrophin‐deficient mouse muscles, female wild‐type MSCs induced more dystrophin‐positive myofibers than male counterparts (Deasy et al., 2007). They also showed that, regardless of the cell sex, MSC‐mediated regeneration was more efficient in female hosts than in males. McHale et al., on the contrary, concluded that no female advantage exists for muscle regeneration when they observed similar absolute CSA of regenerating myofibers between the two sexes after cardiotoxin‐induced injury (McHale et al., 2012). However, the interpretation of the results from this study is confounded by the fact that myofiber size in uninjured females was initially smaller than in males. Hence, their results could also imply that the injured muscles in female mice may have regenerated better and recovered faster to the baseline than males. In addition to this unsettled morphology‐based sex difference in muscle regeneration, no study has compared the function and other properties of regenerating muscles between males and females. In this study, we aimed to address these unresolved gaps by analyzing skeletal muscle size, force, myosin heavy chain (MHC) expression, and fatigability during acute injury‐induced regeneration in male and female mice.

## 2 METHODS

### 2.1 Ethical approval

All animal experiments were approved by the Institutional Animal Care and Use Committee at the University of Illinois at Urbana‐Champaign (protocol #16157 and #19255) in accordance with the Guide for the Care and Use of Laboratory Animals published by the National Institutes of Health (2011, 8th edition). The present study complied with the ARRIVE guidelines 2.0 regarding methods and animal handling. All precautions were taken to minimize animal pain and suffering.

### 2.2 Animals and husbandry

C57BL/6N mice were maintained in cages connected to an EcoFlo ventilation system (Allentown Inc.) in a specific‐pathogen‐free animal facility kept at 23°C on a 12‐h light/dark cycle and received a pellet diet and water ad libitum. Male and female mice were randomly allocated to the various experimental groups and subjected to a muscle injury as described below. Three‐month‐old mice were used in all experiments except for force recovery experiments, where we used both 3‐ and 14‐month‐old mice to expand our key conclusion. Isoflurane was used to anesthetize the animals during all surgical procedures and for euthanasia at the end of the experiments. Any animals that accidentally died before the experiments were concluded were not included in subsequent analyses.

### 2.3 Muscle injury

To induce acute muscle injury and subsequent regeneration, tibialis anterior (TA) muscles were injected with 50 μL of 1.2% (w/v) barium chloride (BaCl_2_) dissolved in saline (Caldwell et al., 1990) and collected 7 and 14 days after injury. BaCl_2_ injection induced nearly 100% of the muscle fibers centrally nucleated in male and female specimens. For muscle fiber CSA and MHC expression, TA muscles of separate mice were injected with 50 μL of saline as baseline controls and collected 21 days after injections to ensure recovery from a minor injury, if any, by needle insertion. For force recovery and fatigability, the contralateral TA muscles of the injured mice were injected with 50 μL of saline to serve as internal controls and analyzed together with the injured muscles. Using internal controls in these experiments is important because muscle force varies widely among different animals with different body/muscle sizes.

### 2.4 Histological analyses

Upon collection, a proximal portion of the TA muscle was cut crosswise and frozen in liquid nitrogen for western blotting. The remaining portion of the muscle was submerged in optimal cutting temperature compound (Tissue‐Tek, Sakura Finetek), frozen in liquid nitrogen–chilled isopentane, and cut in 10 μm thickness at mid‐belly with a Microm HM550 cryostat (Thermo Fisher Scientific). The cross‐sections were placed on microscope slides and subjected to H&E staining. Several H&E‐stained images covering most of the area of the TA section were captured with a 20X dry objective (Fluotar, numerical aperture 0.4; Leica) on a Leica DMI 4000B microscope and analyzed for CSA and minimal Feret's diameter of centrally nucleated regenerating myofibers (approximately 700 on average) using ImageJ (NIH). All procedures were performed by investigators blinded to sample identification.

### 2.5 Western blotting

TA muscle samples were homogenized with a Polytron in ice‐cold buffer containing 20 mM Tris (pH 7.4), 0.3% Triton X‐100, 2 mM EGTA, 2 mM EDTA, 0.1 mM Na_3_VO_4_, 25 mM NaF, 25 mM β‐glycerolphosphate, and 1 × protease inhibitor cocktail (MilliporeSigma, P8240). Protein concentrations of the whole homogenates were measured with the DC protein assay kit (Bio‐Rad). An equal amount of protein from each sample was boiled in Laemmli buffer, resolved on SDS‐PAGE, transferred onto PVDF membrane (MilliporeSigma), blocked with 5% milk in PBS‐T (PBS with 0.5% Tween 20), and probed with the following primary and secondary antibodies: anti‐sarcomere myosin heavy chain (MHC) (clone MF 20; RRID: AB_2147781), anti‐type I MHC (clone BA‐D5; RRID: AB_2235587), anti‐type IIA MHC (clone SC‐71; RRID: AB_2147165), anti‐type IIX MHC (clone 6H1; RRID: AB_1157897), anti‐type IIB MHC (clone BF‐F3; RRID: AB_2266724), and anti‐embryonic MHC (clone F1.652; RRID: AB_528358) from the Developmental Studies Hybridoma Bank (University of Iowa); peroxidase‐conjugated anti‐mouse IgG (Cat# 115‐035‐206) from Jackson Immuno Research Laboratories. After washing in PBS‐T, blots were developed, visualized, and quantified using the SuperSignal West Pico PLUS Chemiluminescent Substrate, an iBright CL1000 Imaging System (both from Thermo Fisher Scientific), and ImageJ (NIH, https://imagej.nih.gov/ij/), respectively.

### 2.6 TA muscle force measurement

Muscle force measurement in regenerating TA muscle was performed in situ using a 1300A Whole‐Animal System (Aurora Scientific) as described previously (You, Singh, et al., 2021). Briefly, the mouse was stabilized on an isothermal stage set at 38°C by inserting a needle through a fixed post and patella tendon. The distal tendon of the TA muscle was connected to the lever arm of the force transducer through a 3‐0 suture line. The muscle was directly stimulated through two electrodes with 0.2‐ms square‐wave pulses at 0.2 mA and adjusted to optimal muscle length where maximal twitch force was produced. The maximum isometric tetanic force was determined in the frequency range of 50–200 Hz with a 300‐ms pulse duration, with each contraction separated by a 1‐minute rest. Throughout the experiments, the exposed TA muscle was kept moist with a warm PBS‐soaked KimWipe.

### 2.7 Muscle fatigability test

TA muscle was set on the force measurement system as above and stimulated every second for 3 min at 150 Hz. After the fatiguing contractions, percentage changes in muscle force and half relaxation time were calculated as indices of muscle fatigability.

### 2.8 Statistics

All values were presented as mean ± SEM with individual data points shown in graphs. The sample size for each experiment was determined based on previous publications and preliminary data. A quantified sample value that deviated more than three times SD from the mean in a given group was removed as an outlier (no more than one outlier was found in any group). Analysis of statistical significance (*p* < 0.05) was performed by a two‐tailed unpaired *t*‐test or two‐way ANOVA using SigmaStat (Systat Software). When comparing the means of groups cross‐classified by within‐subjects (injured vs. contralateral uninjured) and between‐subjects (males vs. females) variables, two‐way mixed ANOVA was used with OriginPro 2021b software (OriginLab) (You & Chen, 2021; You, Kim, et al., 2021). Where significant, *p* values of interaction and main effects from ANOVA analysis were indicated above each graph. All statistical analyses were performed in accordance with the assumptions of the respective tests.

## 3 RESULTS

### 3.1 Effects of injury and sex on muscle size and function

**FIGURE 1:**
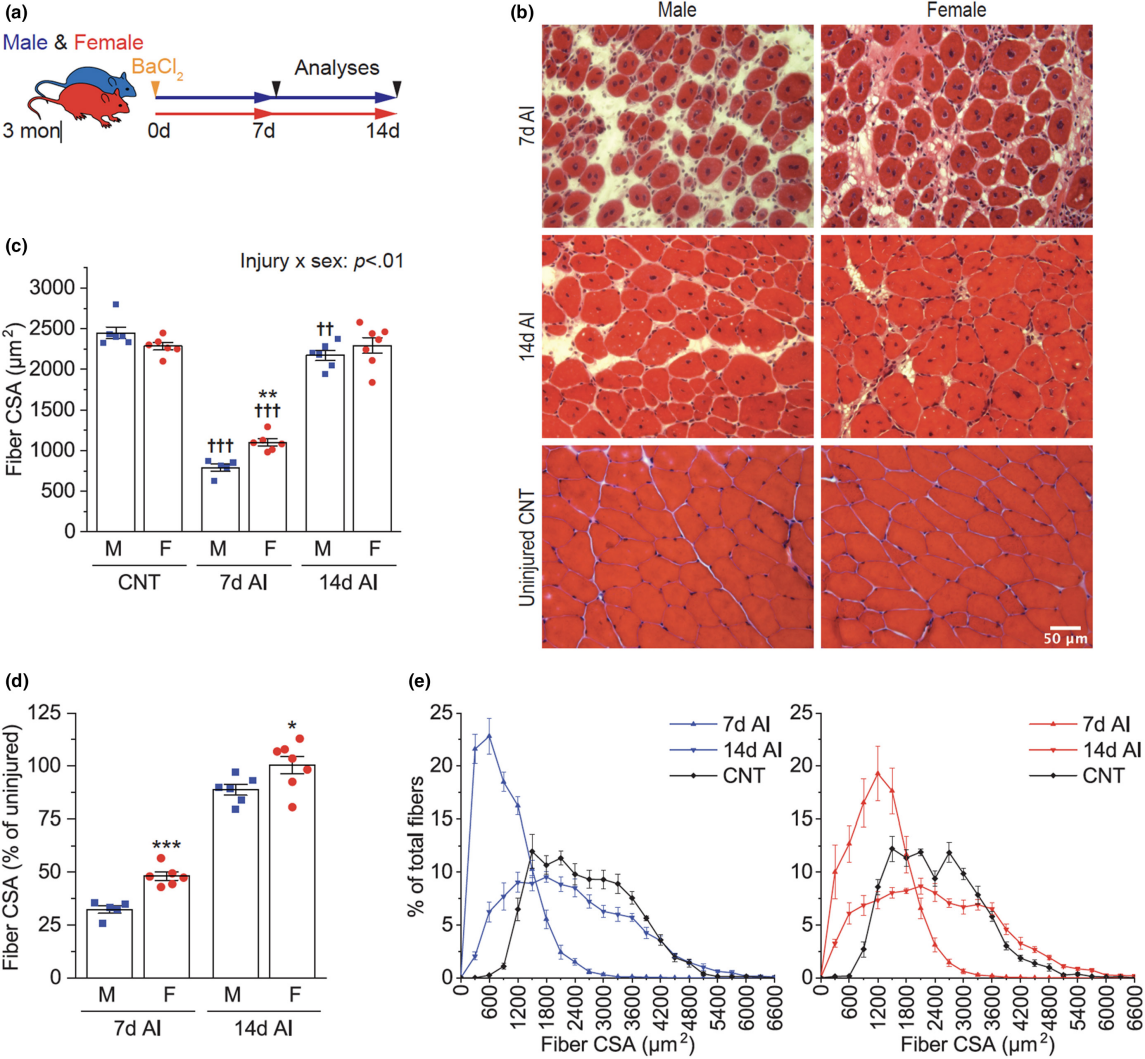
Effects of injury and sex on muscle size. (a) Tibialis anterior muscles of 3‐month‐old male (M) and female (F) mice were injured with BaCl_2_ injection and collected 7 and 14 days after injury (AI). (b) Representative H&E images of cross‐sections of injured and uninjured control (CNT) muscles. (c–e) The cross‐sectional area (CSA) of myofibers was measured from the H&E images (c) and presented as a percentage of uninjured control (d) or on a histogram (e) (*n* = 5–7). Data are presented as mean ± SEM. Statistical significance was determined by two‐way ANOVA followed by Student–Newman–keuls post hoc test (c) or unpaired *t*‐test (d). *p < 0.05, **p < 0.01, ***p < 0.001 versus males at each time point; ^††^ *p* < 0.01, ^†††^ *p* < 0.001 versus CNT.

To examine potential sex differences in skeletal muscle regeneration, we first analyzed the CSA of centrally nucleated regenerating myofibers 7 and 14 days after BaCl_2_‐induced injury (Figure 1a,b). In uninjured conditions, female myofiber size tended to be smaller than male size, but it did not reach statistical significance (*p* = 0.08) (Figure 1c). Despite this trend, the size of regenerating myofibers in female mice was larger than that of male mice 7 days after injury (AI) (Figure 1c). This difference is unlikely due to artifacts such as oblique muscle cuts as similar results were also observed with minimum Feret's diameter (Figure S1). At Day 14 AI, the size of regenerating myofibers was no longer different between male and female mice; however, when compared to the uninjured muscle fibers, male regenerating myofibers were still smaller, while female regenerating myofibers were indistinguishable in size from the baseline (Figure 1c). When the results were expressed relative to the uninjured muscle size, females recovered muscle size faster than males throughout the regeneration process (Figure 1d). Histogram analysis revealed that a substantial number of regenerating myofibers were still smaller than uninjured ones 14 days AI in both male and female mice. However, in females, there was also a population of muscle fibers larger than the uninjured fibers, accounting for the similar average myofiber size between the injured and uninjured muscles in females (Figure 1e).

**FIGURE 2:**
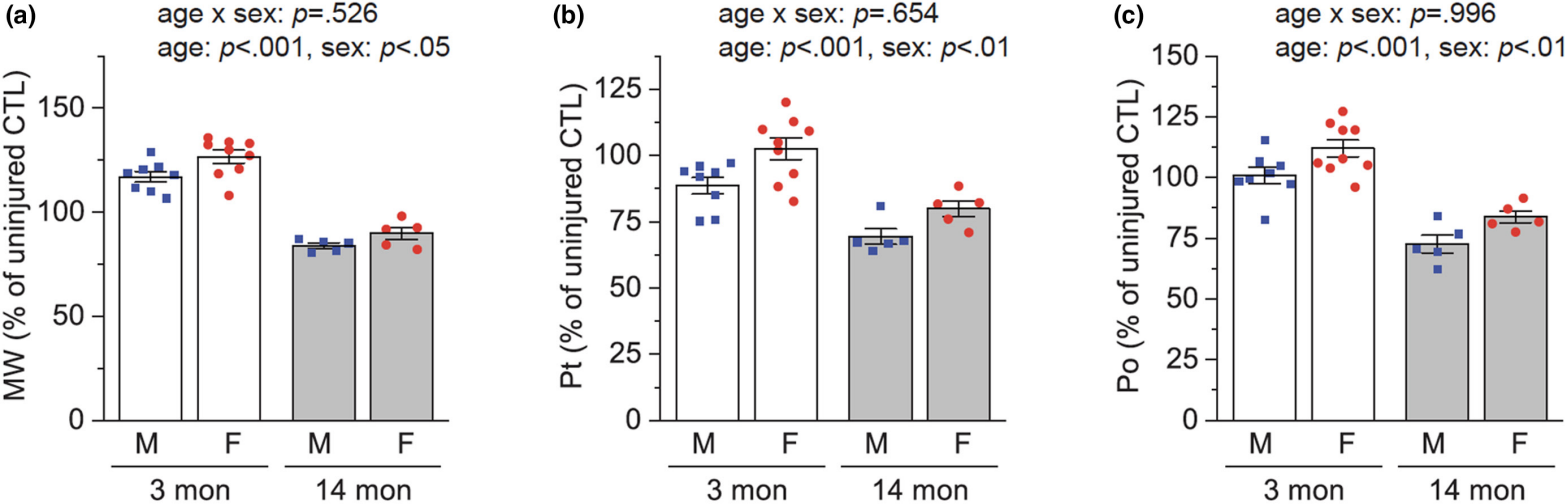
Effects of age and sex on the recovery of injured muscle function. (a–c) Tibialis anterior muscles of 3‐ and 14‐month (mon)‐old male (M) and female (F) mice were injured with BaCl_2_ injection and analyzed 14 days after injury. Muscle weight (MW) (a), maximal isometric twitch force (Pt) (b), and maximal isometric tetanic force (Po) (c) were expressed as a percentage of uninjured contralateral control (CTL) (*n* = 5–9). Data are presented as mean ± SEM. Statistical significance was determined by two‐way ANOVA.

To examine whether sex difference also exists in the recovery of muscle function after injury, we analyzed maximal isometric muscle force at Day 14 AI in males and females. Consistent with the muscle fiber size results, females showed more robust recovery of whole muscle mass (Figure 2a), twitch muscle force (Figure 2b), and tetanic muscle force (Figure 2c) than males. This female‐favored functional muscle regeneration was also observed at 14 months of age (i.e., middle age) when regenerative capacity had already declined (Figure 2a–c), suggesting that the sex difference is independent of the effects of age on muscle regeneration.

### 3.2 Effects of injury and sex on muscle MHC isoform expression

**FIGURE 3:**
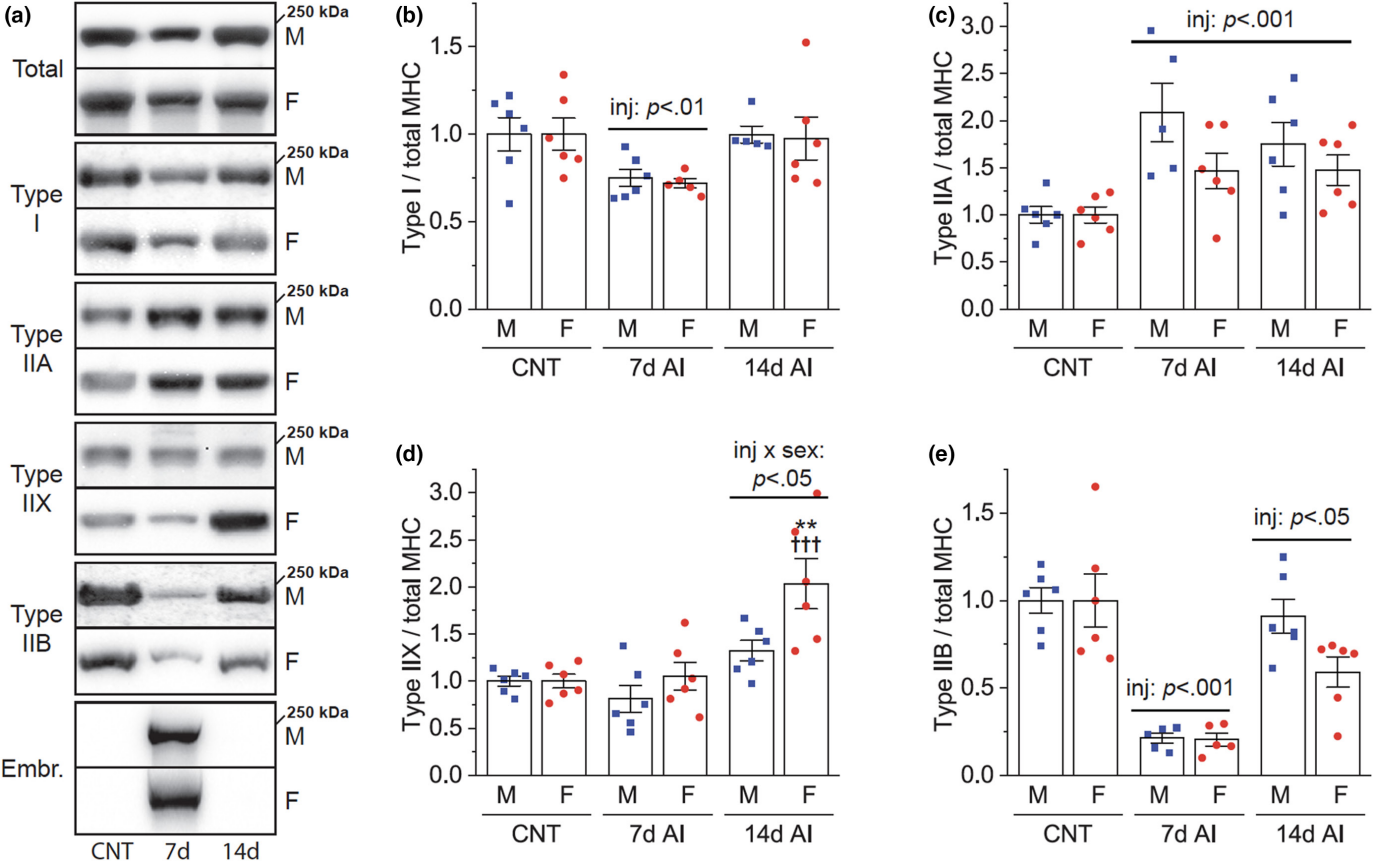
Effects of injury and sex on muscle MHC isoform expression. Tibialis anterior muscles of 3‐month‐old male (M) and female (F) mice were injured with BaCl_2_ injection and collected 7 and 14 days after injury (AI). (a) Various myosin heavy chain (MHC) isoforms were detected by western blotting in injured and uninjured control (CNT) muscles. (b–e) The expression of each MHC isoform was quantified and normalized by total MHC (*n* = 5–6). Data are presented as mean ± SEM. Statistical significance was determined by two‐way ANOVA followed by Student–Newman–keuls post hoc test. The horizontal bars indicate the significant main effects of injury (inj) versus CNT or interaction between injury and sex at the corresponding time point. **p < 0.01 versus males; ^†††^ *p* < 0.001 versus CNT.

We next asked whether sex and/or regeneration influence MHC isoform expression, which is widely used to characterize skeletal muscle properties. By quantifying the relative amounts of individual MHC isoforms after injury (Figure 3a), we found that regenerating muscle exhibits dynamic changes in the expression of MHC isoforms. Specifically, in both males and females, the amount of type I MHC was transiently reduced at Day 7 AI (Figure 3b), while the amount of type IIA MHC increased through 14 days AI (Figure 3c). Interestingly, type IIX MHC increased only in females at Day 14 AI (Figure 3d), indicating a sex‐dependent expression of type IIX MHC. The amount of type IIB MHC was found to be markedly reduced at Day 7 AI (Figure 3e), and it remained lower at Day 14 AI. Although these reductions were not statistically different between males and females, the reduction at Day 14 AI appeared to be more severe in females (Figure 3e), which is likely associated with the female‐specific increase in the amount of type IIX MHC. Overall, these results reveal dynamic remodeling of MHC isoform expression and a shift from type IIB to type IIA/X MHC during regeneration, with sex playing a partial role in this process.

### 3.3 Effects of injury and sex on muscle fatigability and twitch kinetics

**FIGURE 4:**
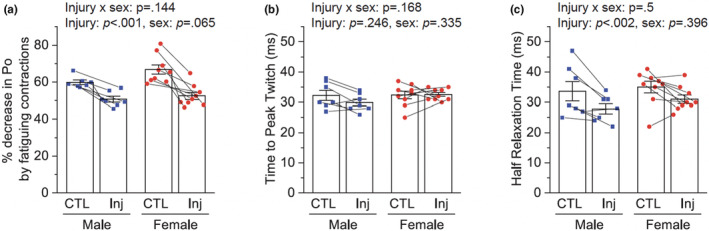
Effects of injury and sex on muscle fatigability and twitch kinetics. Tibialis anterior muscles of 3‐month‐old male (M) and female (F) mice were injured with BaCl_2_ injection and analyzed 14 days after injury. (a) Maximal isometric tetanic force (Po) was measured from injured (Inj) and uninjured contralateral (CTL) muscles before and after fatiguing contractions and expressed as a percentage change (n = 7–9). (b, c) Time to peak twitch (b) and half relaxation time (c) were analyzed from maximal twitch force. Data are presented as mean ± SEM. Statistical significance was determined by two‐way mixed ANOVA followed by Tukey's post hoc test.

Type IIB and IIA MHC isoforms are predominantly expressed in type IIB and less fatigable type IIA muscle fibers, respectively. To investigate the functional relevance of the type IIB to IIA/X MHC transition in regenerated muscles and the role of sex in this event, we tested muscle fatigability at Day 14 AI using the degree of fatiguing contraction‐induced decrease in muscle force. As expected, after 180 times of fatiguing contractions, muscle force was decreased by around 60–70% in uninjured control muscles (Figure 4a). However, this decrease was attenuated in injured and regenerated muscles with no significant sex difference (Figure 4a). Intrigued by these observations, we speculated that muscle contraction and relaxation speed might also be slowed in the regenerated muscles in association with the type IIB to slower type IIA/X MHC isoform shift. However, neither injury nor sex affected the time to peak twitch force, and injury even quickened the half relaxation time regardless of the sex (Figure 4b<c).

## 4 DISCUSSION

A better understanding of sex‐based differences in skeletal muscle regeneration could help develop sex‐specific strategies for regenerative medicine. The current study reveals that skeletal muscle regenerates more efficiently in females than males in mice. This female‐favored regeneration persisted even when the age‐related decline in regenerative capacity had already been initiated. These findings are consistent with the previous study in which female MSCs promoted more efficient regeneration than male MSCs when transplanted into dystrophin‐deficient mouse muscle (Deasy et al., 2007). Considering this previous result, the female‐favored regeneration observed in our study is likely, at least in part, attributed to a sex‐specific cell‐autonomous mechanism. Regarding the recovery to the baseline, our findings are also in line with the study by McHale et al. in which regenerating myofiber CSA in female mice reached the baseline myofiber size faster than in male mice (McHale et al., 2012).

Only a few studies have examined the response of MSCs to muscle damage in human males and females, and whether a sex difference exists in muscle regeneration is uncertain. For instance, by employing step exercise, Imaoka et al. found that eccentric, but not concentric, contractions increased the number of PAX7‐positive MSCs up to 5 days post‐exercise, and no sex difference existed in this event (Imaoka et al., 2015). On the contrary, Fortino et al. found that males had a greater expansion of PAX7‐positive MSCs 2 days following eccentric contractions on the Biodex dynamometer, but they did not find differences in the population of activated MSCs positive for both PAX7 and MyoD (Fortino et al., 2022). While that study advanced the potential role of sex by analyzing activated MSCs, the single time point studied limited the interpretation of the findings. Furthermore, neither of those studies examined MSCs' contribution to damaged muscle fibers, such as fusion or changes in muscle size or function. Although our study reveals apparent sex differences in muscle regeneration in mice, understanding the role of sex in human muscle regeneration will require a more comprehensive characterization of the myogenic response to muscle damage.

Another significant finding in our study is the remodeling of MHC isoform expression and the transition to less fatigable muscle during regeneration. Using immunohistochemical staining and western blotting of fast and slow MHC, Matsuura et al. observed a fast‐to‐slow MHC conversion in the cardiotoxin‐injured regenerating mouse soleus muscle, which led to a conclusion of a fast‐to‐slow myofiber conversion during regeneration (Matsuura et al., 2007). Our western blot analysis of four different MHC isoforms in a different muscle type (TA) injured with barium chloride corroborates the fast‐to‐slow MHC isoform shift during regeneration. This phenomenon is also consistent with our fatigability data, as a higher proportion of slower MHC isoforms is associated with a shift toward less fatigable muscle. However, interestingly, our data regarding twitch kinetics (Figures 4b,c), which showed even faster relaxation speed in the regenerated muscle, do not support another commonly used translation of a fast‐to‐slow MHC isoform shift to a fast‐to‐slow fiber‐type shift. Therefore, our study reveals a unique combination of regenerated muscle properties exhibiting less fatigability and faster twitch speed and suggests a need for caution in interpreting the MHC isoform expression data to define the twitch speed of regenerated muscles.

Our study has some limitations that should be acknowledged. First, our analysis was confined to the TA muscle, which is predominantly composed of type IIX and IIB myofibers. It remains unclear whether the sex differences observed in our study exist in other types of muscle that contain a higher proportion of type I and IIA myofibers. Our method of detecting MHC isoforms through western blotting did not allow for specific fiber typing. Based on the dynamic changes in MHC isoform expression observed in our study, future studies should include fiber typing using immunohistochemical staining of MHC or succinate dehydrogenase to gain a more comprehensive understanding of how regeneration and sex affect muscle fiber types.

## FUNDING INFORMATION

This work was supported by a Muscular Dystrophy Association grant to J.‐S.Y. (#864137) and NIH grants to J.C. (R01AR048914, R56AR048914, and R01GM089771).

## CONFLICT OF INTEREST STATEMENT

None of the authors has any conflicts of interests.

## Supporting information


Figure S1.

## Data Availability

The raw data that support the conclusions of this study will be made available by the authors upon reasonable request.
