# Supplementary material for: Sex differences in skeletal muscle size, function, and myosin heavy chain isoform expression during post‐injury regeneration in mice
Source: Physiol Rep. 2023 Aug 24;11(16):e15791. doi: 10.14814/phy2.15791 (PMC10449603; doi:10.14814/phy2.15791)
Supplement: Supplementary file 1 — Figure S1. [file PHY2-11-e15791-s001.docx]

**
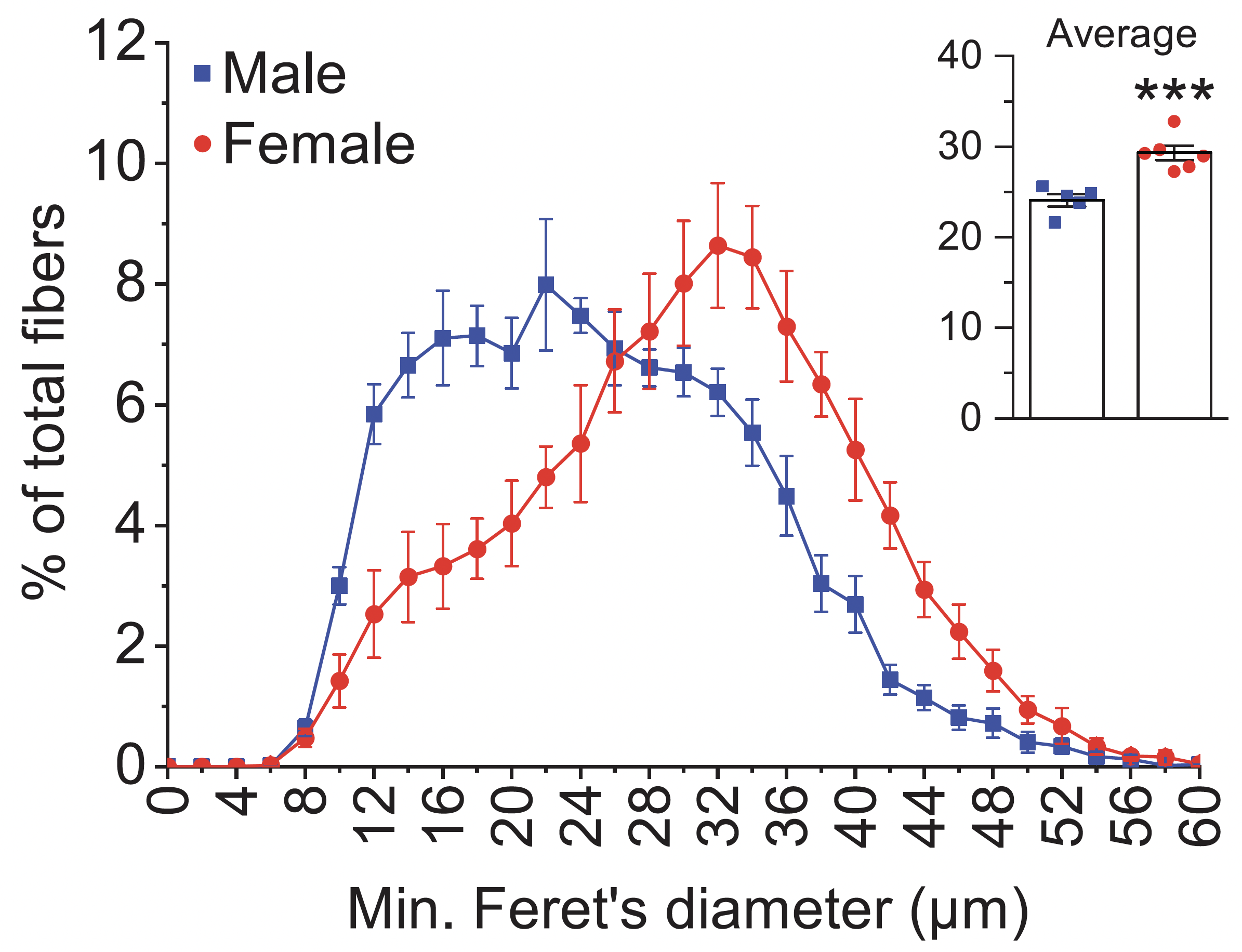
**

**Figure S1. Effects of sex on regenerating muscle size.** Tibialis anterior muscles of 3-month-old male and female mice were injured with BaCl_2_ injection and collected 7 days after injury. Minimum Feret’s diameter of myofibers was measured from the cross sections of the injured muscles (same myofibers as those measured for CSA in Figure 1) and presented on a histogram with an inset showing averaged diameter (n=5-6). Data are presented as mean ± SEM. Statistical significance was determined by an unpaired t-test. ***p<0.01 vs. males.
